# Early Administration of Glutamine Protects Cardiomyocytes from Post-Cardiac Arrest Acidosis

**DOI:** 10.1155/2016/2106342

**Published:** 2016-12-12

**Authors:** Yan-Ren Lin, Chao-Jui Li, Shih-Han Syu, Cheng-Hao Wen, Waradee Buddhakosai, Han-Ping Wu, Cheng Hsu Chen, Huai-En Lu, Wen-Liang Chen

**Affiliations:** ^1^Department of Emergency Medicine, Changhua Christian Hospital, Changhua, Taiwan; ^2^School of Medicine, Kaohsiung Medical University, Kaohsiung, Taiwan; ^3^School of Medicine, Chung Shan Medical University, Taichung, Taiwan; ^4^Department of Emergency Medicine, Chang Gung Memorial Hospital-Kaohsiung Medical Center, Chang Gung University College of Medicine, Kaohsiung, Taiwan; ^5^Department of Public Health, College of Health Science, Kaohsiung Medical University, Kaohsiung, Taiwan; ^6^Bioresource Collection and Research Center, Food Industry Research and Development Institute, Hsinchu, Taiwan; ^7^Department of Biological Science and Technology, National Chiao Tung University, Hsinchu, Taiwan; ^8^Interdisciplinary Graduate Program in Genetic Engineering, Graduate School, Kasetsart University, Bangkhen Campus, Bangkok, Thailand; ^9^Division of Pediatric General Medicine, Department of Pediatrics, Chang Gung Memorial Hospital at Linkou, Kweishan, Taoyuan, Taiwan; ^10^College of Medicine, Chang Gung University, Taoyuan, Taiwan

## Abstract

Postcardiac arrest acidosis can decrease survival. Effective medications without adverse side effects are still not well characterized. We aimed to analyze whether early administration of glutamine could improve survival and protect cardiomyocytes from postcardiac arrest acidosis using animal and cell models. Forty Wistar rats with postcardiac arrest acidosis (blood pH < 7.2) were included. They were divided into study (500 mg/kg L-alanyl-L-glutamine, *n* = 20) and control (normal saline, *n* = 20) groups. Each of the rats received resuscitation. The outcomes were compared between the two groups. In addition, cardiomyocytes derived from human induced pluripotent stem cells were exposed to HBSS with different pH levels (7.3 or 6.5) or to culture medium (control). Apoptosis-related markers and beating function were analyzed. We found that the duration of survival was significantly longer in the study group (*p* < 0.05). In addition, in pH 6.5 or pH 7.3 HBSS buffer, the expression levels of cell stress (p53) and apoptosis (caspase-3, Bcl-xL) markers were significantly lower in cardiomyocytes treated with 50 mM L-glutamine than those without L-glutamine (RT-PCR). L-glutamine also increased the beating function of cardiomyocytes, especially at the lower pH level (6.5). More importantly, glutamine decreased cardiomyocyte apoptosis and increased these cells' beating function at a low pH level.

## 1. Introduction

The survival rate for out-of-hospital cardiac arrest (OHCA) is very low [1–4]. Most sustained return of spontaneous circulation (ROSC) OHCA patients still die from post-cardiac arrest injuries [5–8]. These post-cardiac arrest injuries are critical and systemic reactions, including inflammatory overreactions, failed immune regulation, free-radical attack, and acidosis [2, 9, 10]. Among these injuries, acidosis might start before the event that triggers cardiac arrest (such as a respiratory problem causing respiratory acidosis or infection causing metabolic acidosis), and the severity of the acidosis could become more severe once the circulation collapses (tissue ischemia/reperfusion injury, hypoxia, and free radicals all contribute to acidosis) [6, 9, 11, 12].

The cells of vital organs have been demonstrated to be at risk of apoptosis at low pH levels [13–15]. Furthermore, certain previous studies have reported that early, effective treatment for acidosis might decrease vital organ damage and further increase the survival rate [16, 17]. Therefore, sodium bicarbonate was initially recommended to treat post-cardiac arrest acidosis to restore the acid-base balance, and over the past 30 years, it was even suggested in standard resuscitation guidelines [18, 19]. Unfortunately, recent studies noted major side effects for sodium bicarbonate used for post-cardiac arrest acidosis (including inactivation of simultaneously administered catecholamines, reduction of systemic vascular resistance, hyperosmolality, extracellular alkalosis despite intracellular PCO_2_ excessو and hypernatremia), and it is no longer recommended in new resuscitation guidelines [18, 20, 21]. Therefore, effective and safe medication for treating post-cardiac arrest acidosis is still lacking.

Glutamine, traditionally considered to be a nonessential amino acid, is now considered as conditionally essential following critical illness and sepsis [22–24]. Recently, glutamine was demonstrated to increase ammoniagenesis and gluconeogenesis in the kidney. Excretion of the resulting ammonium ions increased the excretion of acid, whereas the combined pathways also contributed to the production of HCO_3_ (−) ions [25–29]. Therefore, we suspected that glutamine might be a potential medication for treating post-cardiac arrest acidosis. In the present study, we aimed to analyze whether early administration of glutamine could improve survival and protect cardiomyocytes from post-cardiac arrest acidosis using animal and cells models.

## 2. Materials and Methods

### 2.1. Ethics Statement

A total of 43 10-week-old male Wistar rats (301–325 g in weight) obtained from BioLASCO Taiwan Co. Ltd. (Taipei, Taiwan) were used to analyze the* in vivo* treatment effect of glutamine in this study. Before the start of the study, all animals were fasted for 12 hours but given free access to water. The protocol was approved by the Committee on the Ethics of Animal Experiments of Changhua Christian Hospital (Permit Number: CCH-AE-104-005) and adhered to the recommendations of the Guide for the Care and Use of Laboratory Animals of the National Institutes of Health.

### 2.2. Setup of Animal Cardiac Arrest Model: Airways, Intravenous Line, and Measurements of Vital Signs

All rats were anesthetized with isoflurane via inhalation. After short-term inhalation, endotracheal tube (16-gauge polyethylene catheter mounted on a blunt-tipped needle) intubation was performed using the BioLITE Intubation Illumination System®. The rats were ventilated with controlled intermittent positive pressure ventilation (IPPV) (Hallowell EMC Model AWS™) with a tidal volume of 7 mL/kg, a respiratory rate of 80/min, and a fractional inspired oxygen reading of 1.0. A 24-gauge polyethylene catheter (Becton-Dickinson) was advanced into the tail vein for drug administration. Moreover, the cardiac rhythms were measured via Leads I and II using subcutaneous needles (Bio Amp cable and leads, LabTutor® PowerLab, ADInstruments). Blood pressure was measured in the tails of the rats (BP-2000 SERIES II®, noninvasive blood pressure analysis system). The rectal temperature was maintained at 37.0 ± 0.5°C during the experimental period.

### 2.3. Six Minutes of Global Ischemia to Induce Post-Cardiac Arrest Acidosis (pH < 7.2)

Cardiac arrest was induced in all 43 rats by stopping the IPPV and clamping the endotracheal tube to induce asphyxia. Cardiac arrest was confirmed based on an abrupt decrease in systolic arterial pressure to less than 30 mmHg or cardiac rhythms that revealed asystole, ventricular tachycardia/ventricular fibrillation (VT/VF), or pulseless electrical activity (PEA). Immediately after 6 minutes of global ischemia, resuscitation started (blood was also withdrawn for analyzing the pH level). To prevent delay of resuscitation by waiting for pH data, each rat immediately underwent the resuscitation procedures, including (1) mechanical ventilation (100% O_2_, respiratory rate of 60 breaths/min), (2) chest cardiac massage (200 times/min, as performed by a mechanical device), and (3) intravenous epinephrine (0.02 mg/kg). However, if a rat did not reach post-cardiac arrest acidosis (pH < 7.2), it was not included in this study (*n* = 3). Once the rats had their spontaneous circulation restored, the cardiac massage and epinephrine administration were no longer provided. Ultimately, a total of 40 rats that were confirmed to have post-cardiac arrest acidosis and that received resuscitation were included in this study.

### 2.4. Treatment of Rats with Post-Cardiac Arrest Acidosis

All 40 rats were randomly divided into two groups (each *n* = 20), receiving a single administration of 500 mg/kg L-alanyl-L-glutamine (Dipeptiven®, study group) or normal saline (control group) intravenously before resuscitation was started. All rats were treated with the same volume (1 mL) via intravenous injection.

### 2.5. Assessment of Secondary Outcomes:* Sustained ROSC*

In this study, sustained ROSC was defined by spontaneous cardiac rhythm in conjunction with a rise in mean arterial pressure to greater than 50 mmHg for at least 20 minutes [30]. After 30 minutes of unsuccessful cardiopulmonary resuscitation (CPR), resuscitation was stopped, and the animals were declared dead. The rates of sustained ROSC in the study and control groups were recorded.

### 2.6. Assessment of Primary Outcomes:* Duration of Survival*

For each rat that achieved successful resuscitation, hemodynamic measurements (blood pressure, cardiac rhythms) and ventilation were performed for 72 hours maximum. The duration of survival for each rat in the study and control groups was recorded (the maximal observation time was also 72 hours).

### 2.7. Normal Human Cardiomyocyte Preparation (iPSC-Derived Cardiomyocytes)

In this study, induced pluripotent stem cells (iPSCs) were obtained from the Bioresource Collection and Research Center, Food Industry Research and Development Institute (Taiwan), and cultured on Matrigel-coated plates (mTESR medium). The detailed protocols for the iPSC culture and harvesting of iPSC-derived cardiomyocytes adhered to the protocols in previously published studies [31, 32].

### 2.8. Quantitative (Flow Cytometry) and Qualitative (Immunostaining) Analyses

The iPSC-derived cardiomyocytes were checked for transdifferentiation efficiency and cell protein/morphology by quantitative (flow cytometry) and qualitative (immunostaining) analyses, respectively [31]. The cells were detached with Accutase solution (Nalgene) and harvested for quantitative analysis by flow cytometry (BD FACSCanto™ II System). The fixation/permeabilization procedure was performed using the BD Cytofix/Cytoperm kit (BD Pharmingen™). The percentage of cardiomyocytes was calculated by staining with phycoerythrin- (PE-) conjugated anti-human cTnT antibody (BD Pharmingen™). All the samples were stained with the corresponding isotype control (BD Pharmingen™) to ensure specificity. Finally, the data were analyzed with flow cytometry software, and the transdifferentiation efficiency was calculated. In addition, cardiomyocytes were fixed in 4% paraformaldehyde and incubated with antibodies for immunostaining [31, 33]. Antibodies against heart-associated proteins, including anti-human cTnT and NKX2.5 (Human Cardiomyocyte Immunocytochemistry Kit, Life Technologies, Invitrogen), were also used for staining to confirm the morphology of the cardiomyocytes. Finally, the nuclei were stained with DAPI. The immunofluorescence images were visualized with a microscope system, and the cell morphology was recorded at different magnifications.

### 2.9. Exposure to Different pH Levels and RT-PCR Analysis of mRNA Expression of Cell Stress/Apoptosis Markers

The cultured cardiomyocytes were dissociated and equally divided into 5 groups to test the treatment effect of L-glutamine at different pH levels: group A (normal culture medium), group B (pH 6.5 HBSS buffer), group C (pH 6.5 HBSS buffer plus 50 mM L-glutamine), groups D (pH 7.3 HBSS buffer), and group E (pH 7.3 HBSS buffer plus 50 mM L-glutamine). The exposure time in each group was the same (2 hours). Finally, the cardiomyocytes in each group were harvested to analyze the mRNA expression of cell stress and apoptosis markers (caspase-3, Bcl-xL, and p53) using RT-PCR (30 cycles).

### 2.10. Different pH Exposure and Beating Function of Cardiomyocytes

The iPSC-derived cardiomyocytes that we used in this study exhibited regular beating, and the beats per minute (BPM) of the cells could be directly observed. To analyze the treatment effect of L-glutamine on the beating function, cardiomyocytes were equally divided into 5 groups (groups A to E; the conditions of each group are mentioned above). The exposure time in each group was also 2 hours. During treatment, the BPM in each group were recorded (0, 15, 30, 45, 60, and 120 minutes after treatment with L-glutamine). All experiments were independently performed three times.

### 2.11. Data Analysis

A chi-squared test, Fisher's exact test, and one-way ANOVA were used in this study. For the animal study, the descriptive analyses of the independent variables (clinical features) assessed in the study and control rats are reported as percentages and the mean ± standard deviation (SD). The relationships between L-alanyl-L-glutamine and the duration of survival in rats with post-cardiac arrest acidosis were analyzed using survival analyses (Kaplan-Meier curves). Finally, the mean BPM of the cardiomyocytes in each group (groups A to E) were compared using one-way ANOVA at different time points after treatment with L-glutamine. A *p* value < 0.05 was considered statistically significant. All of the analyses were performed using the SPSS statistical package for Windows (Version 15.0, SPSS Inc., Chicago, IL, USA).

## 3. Results

### 3.1. Primary Outcomes of Rats with Post-Cardiac Arrest Acidosis

**Figure 1:**
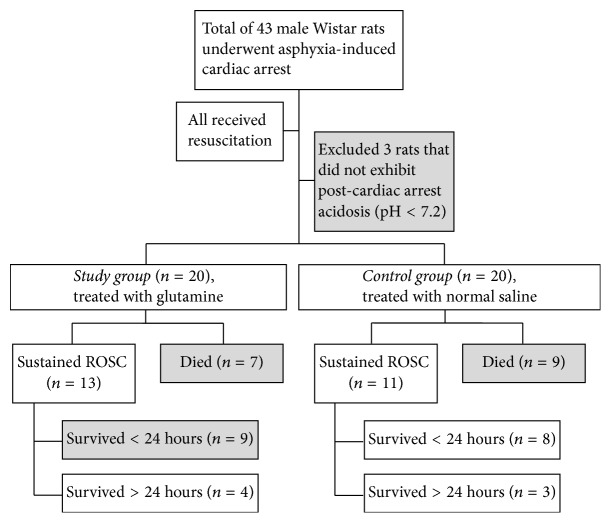
Primary outcomes of rats with post-cardiac arrest acidosis.

The primary and secondary outcomes are shown in Figure 1. The rates of sustained ROSC were 65% (*n* = 13) and 55% (*n* = 11) in the study and control groups, respectively. In all, only 7 rats survived over 24 hours. The clinical features of the rats with post-cardiac arrest acidosis are shown in Table 1. Between the study and control groups (each *n* = 20), the severities of post-cardiac arrest acidosis were nearly equal. The duration of asphyxia (used for inducing cardiac arrest) and the CPR duration were both not significantly different between the two groups. Although the percentages of achievement of sustained ROSC and survival over 24 hours were both higher in the study group than in the control group, the results were statistically significant.

### 3.2. Outcomes of Survival Analysis

**Figure 2:**
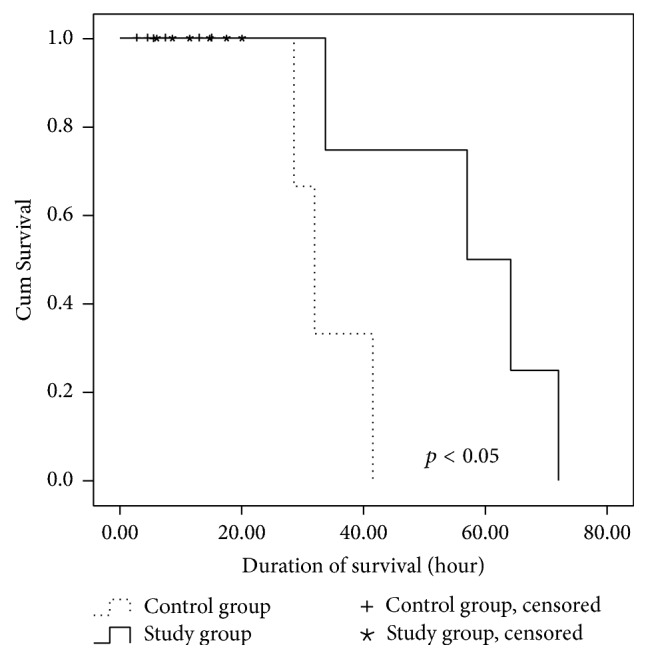
The duration of survival was significantly longer in the study group than in the control group (*p* < 0.05).

The duration of survival was significantly longer in the study group than in the control group (*p* < 0.05) (Figure 2).

### 3.3. Efficiency of Cardiomyocyte Transdifferentiation from iPSCs

**Figure 3:**
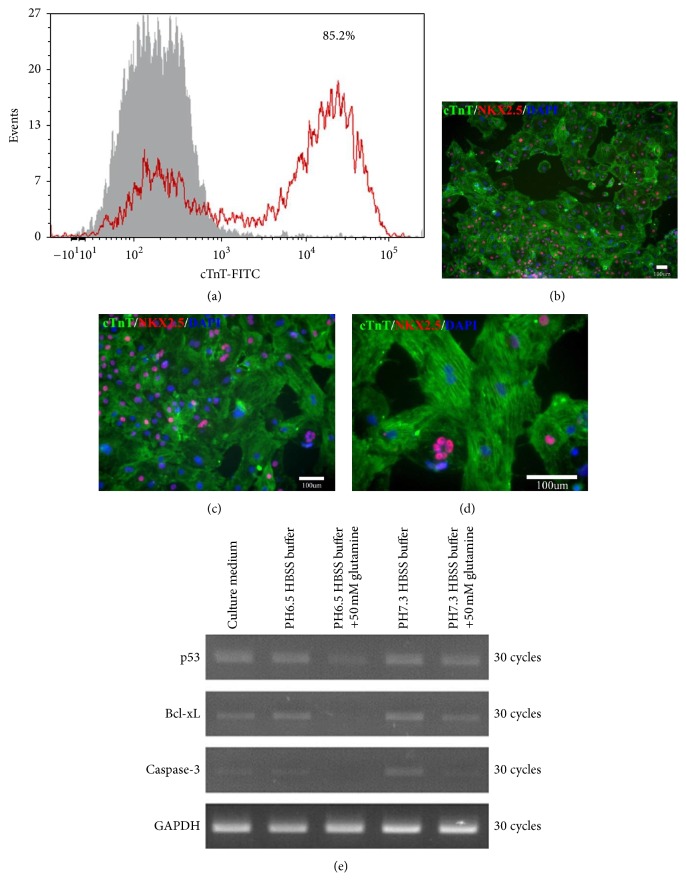
Assessments of both the efficiency of cardiomyocyte transdifferentiation from iPSCs and the outcomes of low pH exposure. (a) Flow cytometry analysis showing that the differentiation efficiency of cTnT+ cells was 85.2%. (b–d) Cardiomyocytes with positive immunostaining for cTnT, NKX2.5, and DAPI at different magnifications. Scale bars: 100 um. (e) RT-PCR results showing that cardiomyocytes treated with 50 mM L-glutamine exhibited decreased expression of caspase-3, Bcl-xL, and p53 in both pH 6.5 and pH 7.3 HBSS buffers. These findings suggest that L-glutamine might protect cardiomyocytes from apoptosis caused by acidosis. Cell groups: A (normal culture medium), B (pH 6.5 HBSS buffer), C (pH 6.5 HBSS buffer plus 50 mM L-glutamine), D (pH 7.3 HBSS buffer), and E (pH 7.3 HBSS buffer plus 50 mM L-glutamine).

The flow cytometry analysis showed that the efficiency of cardiomyocyte transdifferentiation from iPSCs was 85.2% (Figure 3(a)). In this study, these cardiomyocytes presented with functional and regular beating. Immunostaining of these cells revealed that they were positive for cTnT and NKX2.5, which confirmed that the cells that we derived from iPSCs and used in this study were cardiomyocytes (Figures 3(b), 3(c), and 3(d)).

### 3.4. L-Glutamine Might Protect Cardiomyocytes from Apoptosis Caused by Acidosis

In pH 6.5 or pH 7.3 HBSS buffer, the cell stress (p53) and apoptosis (caspase-3, Bcl-xL) markers exhibited obviously lower expression in cells treated with 50 mM L-glutamine than in those without treatment with L-glutamine (based on 30 cycles of RT-PCR) (Figure 3(e)). These findings suggest that L-glutamine might protect cardiomyocytes from apoptosis caused by acidosis.

### 3.5. L-Glutamine Increases the Beating Function of Cardiomyocytes, Especially under Lower pH Conditions

**Figure 4:**
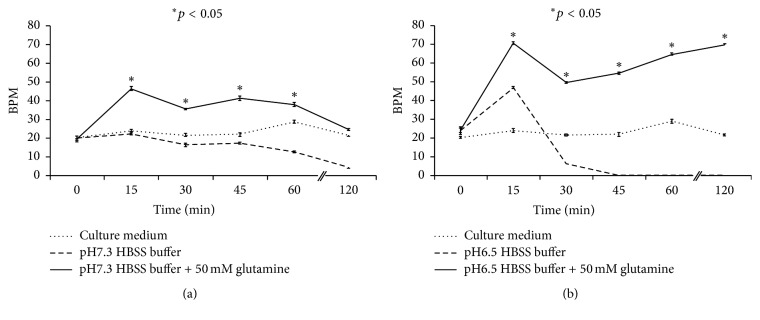
L-glutamine increased the beating function of cardiomyocytes, especially under lower pH conditions. (a) The mean BPM of cardiomyocytes was significantly higher among cells treated with 50 mM L-glutamine in pH 7.3 HBSS buffer than among those that were not treated with L-glutamine. (b) Cardiomyocytes were nearly not beating from the 45th minute after pH 6.5 exposure, but additional exposure to 50 mM L-glutamine could maintain and increase the mean BPM of the cells. One-way ANOVA was used at different time points after treatment with L-glutamine. BPM: beats per minute.

The cardiomyocytes (derived from iPSCs and with beating function) were treated with normal culture medium or pH 7.3 or 6.5 HBSS buffer (with or without 50 mM L-glutamine) for 120 minutes. During this period, the mean BPM values were 23.1 ± 0.8 (culture medium), 15.7 ± 0.9 (pH 7.3 HBSS buffer), 34.4 ± 1.0 (pH 7.3 HBSS buffer with 50 mM glutamine), 12.8 ± 0.6 (pH 6.3 HBSS buffer), and 55.4 ± 0.9 (pH 6.3 HBSS buffer with 50 mM glutamine) (Figure 4). Generally, the mean BPM was significantly higher among cells treated with 50 mM L-glutamine in pH 7.3 or pH 6.5 HBSS buffer than among those that were not treated with L-glutamine. The cardiomyocytes were nearly not beating from the 45th minute after pH 6.5 exposure, but additional exposure to 50 mM L-glutamine could maintain and even increase the mean BPM of the cells.

## 4. Discussion

The primary outcomes of this animal study of cardiac arrest demonstrated that rats treated with early glutamine survived longer than those without glutamine (a positive finding of the survival analysis). Four major explanations might account for this finding.

Firstly, glutamine might effectively control systemic post-cardiac arrest injuries. Severe inflammatory reactions (complement activation and IL-1, IL-6, IL-8, and IL-10 elevation), blood coagulation, platelet activation with formation of thromboxane A2, alteration of soluble E-selectin (sE-selectin) and P-selectin (sP-selectin), and whole-body ischemia/reperfusion injury, which occur in sepsis, also occur in post-cardiac arrest injuries [6, 9, 34, 35]. Since glutamine is well known as a treatment for sepsis (i.e., due to tissue protection, anti-inflammatory/immune reactions, preservation of tissue metabolic functions, and antioxidant activity/attenuation of inducible nitric oxide synthase expression) [36–39], we believed that early glutamine administration would also be beneficial to patients in the postresuscitation period.

Secondly, we suspect that glutamine can indirectly improve acidosis by increasing ammoniagenesis in the kidney. Several previous studies focused on the acid-base balance reported that the renal proximal tubule could obviously increase the uptake and catabolism of glutamine during acidosis [26–29]. Moreover, the increased catabolism of glutamine triggers ammoniagenesis. Excretion of the resulting ammonium ions facilitates the excretion of acid, whereas the combined pathways accomplish the production of HCO_3_ (−) ions, which enter the plasma to restore the acid-base balance [29, 40]. One study further noted that expression of the glutamine transporter Slc38a3 increased in the kidney during metabolic acidosis [25]. Therefore, we suspect that the acid-base balance could be best restored by glutamine supplementation in the early postresuscitation period.

Thirdly, glutamine might directly decrease the apoptosis of cardiomyocytes at a low pH level. In our in vitro study, the expression of caspase-3 and Bcl-xL in cardiomyocytes was obviously decreased following early treatment with glutamine. Clinically, cardiovascular events (i.e., arrhythmia, contraction force dysfunction) might be induced by acidosis [41–43]. Furthermore, acidosis can cause cardiomyocytes to undergo apoptosis via caspase-12/caspase-3 activation (by endoplasmic reticulum (ER) stress, Ca^2+^ leakage) or the mediating effect of BNIP3 (Bcl-2 family) [44–46]. Since glutamine has been demonstrated to have a treatment effect on ER stress [47, 48], we suspect that the post-cardiac arrest acidosis-induced cardiomyocyte apoptosis could be improved by early glutamine administration.

Finally, although several studies reported that glutamine might recover the contractile function of the heart following ischemia/reperfusion injury [49, 50], the treatment effect on cardiomyocytes at a low pH level was still not clear. In the present study, we found that the beating function of cardiomyocytes was obviously increased following treatment with glutamine, especially at a low pH level. We suspected that the reasons for this finding might be cell stress (acid stress, with more acid leading to more stress) and a potential recovery effect on cardiomyocytes at a low pH level.

## 5. Limitations

There were certain limitations to this study. Firstly, the causes of post-cardiac arrest acidosis are complex, and the detailed mechanisms involved were not analyzed in this study. Secondly, the only recorded outcomes for the rats that achieved sustained ROSC were the rate of sustained ROSC and the duration of survival; neurologic evaluations were not performed. Thirdly, although glutamine was demonstrated to reduce ER stress and injury to cardiomyocytes presenting cell apoptosis, the detailed mechanisms involved were not clarified. Finally, hypoxia was not considered in the cell model analysis.

## 6. Conclusion

In conclusion, early administration of glutamine increased the duration of survival in the animal model of post-cardiac arrest acidosis. More importantly, glutamine decreased cardiomyocyte apoptosis and increased these cells' beating function at a low pH level.

## Figures and Tables

**Table 1:** Clinical features of rats with post-cardiac arrest acidosis.

	Total of 40 rats with post-cardiac arrest acidosis		
	Study group (*n* = 20)	Control group (*n* = 20)	
	Number (%)	Number (%)	*p* value
Initial blood pH level (mean ± SD)	7.056 ± 0.091	7.058 ± 0.088	0.958
Duration of asphyxia^*∗*^ (mean ± SD) (min)	13.1 ± 3.8	12.4 ± 3.9	0.537
Duration of CPR (mean ± SD) (min)	11.7 ± 4.6	11.5 ± 4.5	0.938
Sustained ROSC	13(65)	11(55)	0.374
Survival over 24 hours	4(20)	3(15)	0.500

^*∗*^Asphyxia performed to induce cardiac arrest. CPR: cardiopulmonary resuscitation. ROSC: return of spontaneous circulation.
